# Enlarged Perivascular Spaces in the Basal Ganglia Independently Related to Gait Disturbances in Older People With Cerebral Small Vessel Diseases

**DOI:** 10.3389/fnagi.2022.833702

**Published:** 2022-06-22

**Authors:** Shuna Yang, Xuanting Li, Wenli Hu, Wei Qin, Lei Yang

**Affiliations:** Department of Neurology, Beijing Chaoyang Hospital, Capital Medical University, Beijing, China

**Keywords:** cerebral small vessel diseases, enlarged perivascular spaces, Virchow–Robin spaces, gait, elderly individuals

## Abstract

**Background and Objective:**

Gait disturbances are common in older people and are associated with adverse consequences, e.g., falls and institutionalization. Enlarged perivascular spaces in the basal ganglia (BG-EPVS) are considered an magnetic resonance imaging (MRI) marker of cerebral small vessel diseases (CSVD). However, the consequences of BG-EPVS are largely unknown. Previous studies showed that other CSVD markers were related to gait disturbances. However, the relation between BG-EPVS and gait performance is unclear. Therefore, we aimed to explore the relation between BG-EPVS and gait performance in elderly individuals.

**Methods:**

We recruited older people with CSVD in the Neurology Department of our hospital from December 1, 2020 to October 31, 2021. Participants with BG-EPVS > 20 on the unilateral side of the basal ganglia slice containing the maximum number were classified into the BG-EPVS group (*n* = 78), and the rest were classified into the control group (*n* = 164). Quantitative gait parameters and gait variability were provided by the Intelligent Device for Energy Expenditure and Activity (IDEEA; MiniSun, United States) gait analysis system. Semiquantitative gait assessment was measured with the Tinetti test. Point-biserial correlation and multivariate linear regression analysis were performed to investigate the association between BG-EPVS and gait performance.

**Results:**

The BG-EPVS group had a slower gait speed and cadence, shorter stride length, longer stance phase percentage, smaller pre-swing angle and footfall, and lower Tinetti gait test and balance test scores compared with those in the control group (*P* < 0.05). There were no statistical differences in stride length variability and stride time variability between the two groups (*P* > 0.05). A correlation analysis showed that BG-EPVS were negatively related to gait speed, cadence, stride length, pre-swing angle, and footfall (γ_*range*_ = −0.497 to −0.237, *P* < 0.001) and positively related to stance phase percentage (γ = 0.269, *P* < 0.001). BG-EPVS was negatively related to the score of the Tinetti gait test (γ = −0.449, *P* < 0.001) and the balance test (γ = −0.489, *P* < 0.001). The multiple linear regression analysis indicated that BG-EPVS was an independent risk factor for gait disturbances and poor balance after adjusting for confounders, including other CSVD markers.

**Conclusion:**

Large numbers of BG-EPVS were independently related to gait disturbances in older people with CSVD. This finding provides information about the consequences of BG-EPVS and risk factors for gait disturbances.

## Introduction

Gait disturbances are a major issue in older people because they are prevalent and related to adverse consequences, such as falls, institutionalization, and death (Verghese et al., 2006; Abellan van Kan et al., 2009). There is an important clinical significance in exploring the risk factors for and mechanisms underlying gait disturbances in older people. Normal gait control is a complex function that depends on the coordination of multiple brain regions, including cortical, subcortical, and spinal hubs (de Laat et al., 2012; Kim et al., 2016). The basal ganglia refers to a group of important subcortical strcutures related to the control of normal gait, balance, and falls. The motor circuit of the basal ganglia is important for the selection and suppression of movement, the execution of automatic actions, and the scaling of motor outputs (MacKinnon, 2018). Movement initiation and anticipatory adjustments of ongoing motion are achieved by cortical gait control. Automatic gait control is achieved by projections downstream of the mesencephalic locomotor region (MLR) and the pedunculopontine nucleus (PPN) to spinal central pattern generators that produce and modulate basic bipedal locomotor patterns. Tracts descending from the cortical areas project *via* the basal ganglia loop to adjust the activity of MLR/PPN through GABA-ergic inhibitory outputs of the pars reticulata of the substantia nigra (Wagner et al., 2022).

Perivascular spaces, or Virchow–Robin spaces, are compartments surrounding the small cerebral penetrating vessels, serving as a protolymphatic system that plays an important role in interstitial fluid and solute clearance in the brain (Gouveia-Freitas and Bastos-Leite, 2021). Perivascular spaces dilate with the accumulation of interstitial fluids. Enlarged perivascular spaces, visible with magnetic resonance imaging (MRI), appear as punctate or linear signal intensities similar to cerebrospinal fluid (CSF) on all MRI sequences (Rudie et al., 2018). Enlarged perivascular spaces in the basal ganglia (BG-EPVS) are recognized as an MRI marker of cerebral small vessel diseases (CSVD) (Wardlaw et al., 2013). However, the consequences of BG-EPVS are largely unknown. Although they are generally considered clinically silent, some studies explored the association between EPVS and cognitive disturbances (Zhu et al., 2010; Jie et al., 2020). However, the results were not consistent across the studies. Whether the association between EPVS and cognitive impairment is attributed to a direct effect of EPVS or to the accompanying CSVD markers, such as white matter hyperintensities (WMH), cerebral microbleeds (CMB), and lacunes, remains incompletely understood.

Previous studies showed that WMH, CMB, and lacunes were related to gait disturbances (Stijntjes et al., 2016; van der Holst et al., 2018; Cannistraro et al., 2019). However, the relation between BG-EPVS and gait performance is unclear. We speculated that the presence of many BG-EPVS might be associated with gait disturbances by disrupting the function of the basal ganglia. Therefore, we explored the association between BG-EPVS and gait performance after adjusting for WMH, CMB, and lacunes in older individuals.

## Materials and Methods

### Study Subjects

The study was a cross-sectional study. We reviewed the medical records of older patients with CSVD and without acute cerebral infarction who were admitted to the Neurology Department of Beijing Chaoyang Hospital affiliated with Capital Medical University from December 1, 2020 to October 31, 2021. CSVD was defined as the presence of WMH, CMB, and/or lacunes of presumed vascular origin on brain MRI. Patients were selected for participating in the study according to the following inclusion and exclusion criteria. The inclusion criteria were the following: (1) aged 60 or over and (2) agreed to participate in the study. The exclusion criteria were the following: (1) dementia including Alzheimer’s disease, frontotemporal dementia, or dementia with Lewy bodies; (2) Parkinson’s diseases (PD) and Parkinson’s plus syndromes, including multiple system atrophy, cortical basal degeneration, and progressive supranuclear palsy; (3) history of severe stroke (the largest diameter of lesion size > 20 mm) that caused difficulties and inaccurate assessments of CSVD MRI markers or large-vessel cerebrovascular diseases defined as internal carotid, middle cerebral, or basilar intracranial artery stenosis > 50%; (4) lacunar syndrome within 6 months after the event to avoid acute effects on the outcomes, or the patients with stroke sequelae 6 months after cerebral infarction onset; (5) traumatic, toxic or infectious brain injury, brain tumor or brain metastases, or non-CSVD-rated white matter lesions, e.g., multiple sclerosis and irradiation induced gliosis; (6) inability to walk for 30 m unaided; (7) could not finish the tests because of prominent visual, hearing, or language impairments or psychiatric disease; (8) conditions not related to CSVD that affected gait (e.g., joint fusion, severe arthritis, joint replacement, or lumbar spondylopathy); (9) heart failure, myocardial infarction, or angina pectoris disorders during the previous 3 months or severe nephrosis or liver disease with a life expectancy of < 6 months; and (10) MRI contraindications, known claustrophobia, or the patient’s head moved during the MRI, resulting in poor imaging quality that affected the CSVD assessment.

**FIGURE 1:**
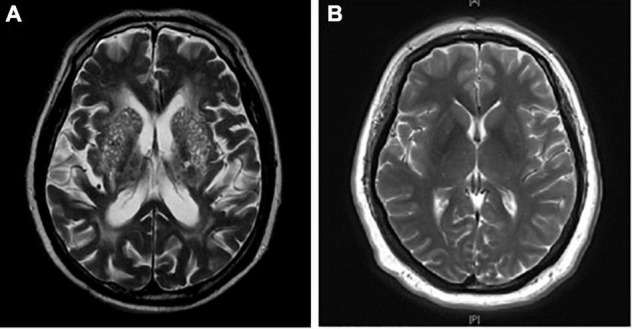
Enlarged perivascular spaces in basal ganglia (BG-EPVS) group and control group. **(A)** Patients with EPVS > 20 on the unilateral side of the basal ganglia slice containing the maximum number were defined as the BG-EPVS group. **(B)** Patients with EPVS ≤ 20 on the unilateral side of the basal ganglia slice containing the maximum number were defined as the control group.

All enrolled patients were divided into two groups according to the number of BG-EPVS on axial T2-weighted images. Patients with BG-EPVS > 20 on the unilateral side of the basal ganglia slice containing the maximum number were classified into the BG-EPVS group (Figure 1A); otherwise, the patients were classified into the control group (Figure 1B). The cutoff > 20 EPVS was used as a high grade, as in previous studies (Doubal et al., 2010).

### Ethical Standards Statement

The study was approved by the Ethics Committee of Beijing Chaoyang Hospital Affiliated with Capital Medical University and was conducted in accordance with the Declaration of Helsinki. All participants provided informed written consent.

### Demographic and Clinical Assessments

The following data were collected: age, sex, body mass index (BMI), past medical history, including hypertension, diabetes mellitus, hyperlipidemia, and stroke, and smoking and alcohol consumption. All blood samples were collected in the morning after an overnight fast and sent to the clinical laboratory of our hospital for the measurement of serum indices, including total cholesterol (TC), triglyceride, high-density lipoprotein (HDL), low-density lipoprotein (LDL), hemoglobin A1c (HbA1c), blood urea nitrogen (BUN), and creatinine.

### Magnetic Resonance Imaging Examinations and Assessments of BG-EPVS, White Matter Hyperintensities, Cerebral Microbleeds, and Lacunes

Using a 3 T MRI scanner (Prisma; Siemens AG, Erlangen, Germany), MRI was performed in the Radiology Department of our hospital. The standardized MRI sequences included T1-weighted, T2-weighted, diffusion-weighted imaging, fluid-attenuated inversion recovery, and susceptibility-weighted imaging.

Imaging markers of CSVD, including BG-EPVS, WMH, CMB, and lacunes, were defined according to the Standards for Reporting Vascular Changes on Neuroimaging criteria described previously (Wardlaw et al., 2013). The number of BG-EPVS was assessed in the basal ganglia slice containing the maximum number of EPVS. Periventricular and deep WMH were evaluated separately and summed to obtain Fazekas scores. A detailed description of the Fazekas scale has been previously published (Fazekas et al., 1987). The numbers of CMB and lacune were counted.

Assessments of BG-EPVS, WMH, CMB, and lacunes were performed by two experienced neurologists blinded to the clinical data. Random scans of 50 individuals were independently examined by the two experienced neurologists blinded to the readings of the other. The intra-rater agreement for the ratings of BG-EPVS, WMH, CMB, and lacunes was assessed on a random sample of 50 individuals, with a 1-month interval between the first and second readings. The k statistics of the intra-rater and interrater agreements were 0.80 and 0.98, indicating good reliability. Disagreement was resolved by discussing it with the other co-authors.

### Gait Measurement

Quantitative gait analysis was performed with the Intelligent Device for Energy Expenditure and Activity (IDEEA; MiniSun, United States), which had excellent test-retest reliability and validity (Gorelick et al., 2009; de la Cámara et al., 2019). The IDEEA was equipped with Windows-based software that analyzed the gait data and provided gait parameters including gait speed, cadence (number of steps per minute), stride length (the distance between the heel points of two consecutive footprints of the same foot), stance phase percentage, footfall (neuromuscular and skeletal control of limbs during the end of swing phase), and pre-swing angle (the foot-ground angle at the moment of toe-off). Gait variability included stride length variability and stride time variability, which reflected the magnitude of stride-to-stride fluctuations. Gait variability was calculated as the coefficient of variation as a percentage [(standard deviation of parameter/mean of parameter) × 100%]. The participants were instructed to walk 8 m wearing the device at their usual gait speed on low-heeled shoes before the formal test. The formal test of walking 8 m was then performed.

Semiquantitative gait assessment was measured using the Tinetti test with 17 items: nine for balance (score 0–16) and eight for gait (score 0–12) with a maximum score of 28 (de Laat et al., 2010). The Tinetti test was independently scored by two experienced neurologists blinded to the clinical data when the participants walked the 8 m; the two scores were averaged. Interrater reliability was calculated. The k coefficient of the interrater agreement was 0.96, indicating good reliability.

### Statistical Analysis

Continuous variables were summarized as mean values ± *SD* or median (interquartile range) according to whether the data conformed to a normal distribution. Categorical variables were presented as absolute numbers and percentages. Continuous variables with both normal distributions and homogeneity of variance were compared using the Student’s *t*-test, whereas were compared using the Wilcoxon rank-sum test. Bonferroni correction was adopted to reset the *p*-value. The Chi-squared test was used for the comparison of categorical variables. Point-biserial correlation analysis was adopted to analyze the correlation between BG-EPVS (a binary variable) and gait parameters (continuous variables). The relationship between BG-EPVS and gait parameters was assessed with the point-biserial correlation coefficient. Partial correlation analysis and multivariate linear regression analysis were performed to analyze whether BG-EPVS was independently related to gait performance after adjusting for confounding factors. Analysis was performed with statistical product and service solutions 21.0 (SPSS 21.0, IBM, United States), and statistical significance was accepted at *P* < 0.05.

## Results

### Participants’ General Clinical Characteristics

A total of 354 elderly patients with CSVD and without acute cerebral infarction during the study period were identified. Among them, we excluded 43 patients who had a history of severe stroke and large-vessel diseases, 5 patients who had been diagnosed with PD, 36 patients who had severe arthritis, joint fusion, lumbar spondylopathy, or visual impairment that affected their walking, and 3 patients whose imaging quality was not high enough to assess markers of CSVD. The other 25 patients did not agree to participate in the study. Ultimately, 242 elderly patients were enrolled in the study and performed the tests, among which 78 patients were classified into the BG-EPVS group and 164 patients were classified into the control group according to the number of BG-EPVS.

The general clinical characteristics of all participants, the BG-EPVS group, and the control group are presented in Table 1. The mean age of all participants was 71 ± 7.7 years old and 141 (58.26%) of them were men. Those in the BG-EPVS group were older than those in the control group. The proportions of those of the male sex, with hypertension, and a history of stroke in the BG-EPVS group were higher than those in the control group. Those in the BG-EPVS group had higher levels of blood creatinine. There were no statistical differences between the BG-EPVS and control groups in BMI and the proportions of the following: smoking and alcohol, history of diabetes and hyperlipidemia, and the other collected clinical characteristics. Considering the imaging characteristics, the BG-EPVS group had more serious WMH, more lacunes, and CMB (*P* < 0.001). The detailed statistical results are shown in Table 1.

**TABLE 1:** General clinical characteristics of participants.

	All participants	BG-EPVS group	Control group	T or Z or Chi-square value	*P*-value
*n*	242	78	164	–	–
Age*^a^*, years	71 ± 7.7	75 ± 8.0	70 ± 7.0	-5.305	< 0.001
Men*^c^*, *n* (%)	141 (58.26)	57 (73.08)	84 (51.22)	10.385	0.001
BMI*^a^*, kg/m^2^	25 ± 3.7	25 ± 3.5	25 ± 3.8	-0.065	0.948
Smoking*^c^*, *n* (%)	77 (31.82)	30 (38.46)	47 (28.66)	2.342	0.126
Alcohol*^c^*, *n* (%)	43 (17.77)	16 (20.51)	27 (16.46)	0.593	0.441
Hypertension*^c^*, *n* (%)	170 (70.25)	62 (79.49)	108 (65.85)	4.701	0.030
Diabetes*^c^*, *n* (%)	65 (26.86)	27 (34.62)	38 (23.17)	3.524	0.060
Hyperlipidemia*^c^*, *n* (%)	89 (36.78)	29 (37.18)	60 (36.59)	0.008	0.929
Stroke*^c^*, *n* (%)	54 (22.31)	30 (38.46)	24 (14.63)	17.312	< 0.001
TC*^a^*, mmol/L	4.4 ± 0.99	4.3 ± 1.00	4.5 ± 0.99	1.091	0.276
Triglyceride*^a^*, mmol/L	1.6 ± 0.92	1.6 ± 0.94	1.6 ± 0.90	0.088	0.930
HDL*^a^*, mmol/L	1.1 ± 0.30	1.1 ± 0.29	1.1 ± 0.31	1.036	0.301
LDL*^a^*, mmol/L	2.8 ± 0.96	2.7 ± 0.98	2.8 ± 0.94	0.907	0.365
HbA1*^a^*, %	6.3 ± 1.27	6.6 ± 1.37	6.2 ± 1.21	-1.973	0.051
BUN*^a^*, mmol/L	5.7 ± 1.72	6.0 ± 1.78	5.6 ± 1.68	-1.705	0.090
Creatinine*^a^*, μmol/L	70 ± 23.2	75 ± 25.3	67 ± 21.6	-2.412	0.017
WMH*^b^*, fazekas score	3 (2–4)	4 (3–5)	2 (2–3)	-8.195	< 0.001
Lacune*^b^*, *n*	0 (0–2)	2 (0–4)	0 (0–1)	-7.546	< 0.001
CMB*^b^*, *n*	0 (0–1)	1 (1–5)	0 (0–1)	-4.705	< 0.001

*BMI, body mass index; TC, total cholesterol; HDL, high-density lipoprotein; LDL, low-density lipoprotein; HbA1c, hemoglobin A1c; BUN, blood urea nitrogen; WMH, white matter hyperintensities. ^a^Continuous variables with normal distribution were expressed as mean values ± standard deviation and were compared with the Student t-test.*

*The statistic value was T. ^b^Continuous variables with non-normally distributions were expressed as median (interquartile range) and compared with the Wilcoxon rank-sum test. The statistic value was Z. ^c^Categorical variables were presented as absolute numbers and percentages and compared with the Chi-squared test. The statistic value was the Chi-square value.*

### Association Between BG-EPVS and Gait Performance

Table 2 shows the gait parameters and statistical results of the BG-EPVS and control groups. Compared with the control group, the BG-EPVS group had a slower gait speed and cadence, shorter stride length, longer stance phase percentage, smaller pre-swing angle, and footfall. The scores of the Tinetti gait test and balance test were lower in BG-EPVS than those in the control group. Statistical differences were retained after Bonferroni correction.

**TABLE 2:** Gait parameters of the enlarged perivascular spaces in the basal ganglia (BG-EPVS) group and the control group.

Gait parameters	BG-EPVS group	control group	*T*- or *Z*-value	*P*
Gait speed*^a^*, m/min	41.4 ± 14.60	52.7 ± 12.84	6.059	< 0.001
Cadence*^a^*, steps/min	99 ± 14.8	106 ± 13.1	2.170	< 0.001
Stride length*^a^*, m	0.8 ± 0.21	1.0 ± 0.17	7.335	< 0.001
Stance phase percentage*^a^*, %	63.6 ± 6.29	59.3 ± 7.68	-3.300	0.001
Foot fall*^a^*, G	2.1 ± 0.80	3.0 ± 0.81	5.207	< 0.001
pre-swing angle*^a^*, °	15.3 ± 6.49	22.6 ± 8.49	5.059	< 0.001
Tinetti gait test*^b^*, sore	11 (9–12)	12 (12–12)	-7.255	< 0.001
Tinetti balance test*^b^*, sore	15 (11–16)	16 (16–16)	-7.539	< 0.001

*^a^Continuous variables with normal distribution were expressed as mean values ± standard deviation and were compared with Student t-test. The statistic value was T. ^b^Continuous variables with non-normally distributions were expressed as median (interquartile range) and compared with the Wilcoxon rank-sum test. The statistic value was Z.*

Point-biserial correlation analysis showed that BG-EPVS were negatively related to gait speed, cadence, stride length, pre-swing angle, and foot control and positively related to stance phase percentage (Table 3). BG-EPVS was negatively related to the scores of the Tinetti gait test and the balance test. We further conducted a partial correlation analysis to adjust for the confounding factors. The negative correlation between BG-EPVS and gait performance and balance did not change even after adjusting for the confounders (Table 3).

**TABLE 3:** Results of correlation analysis between gait performance and BG-EPVS.

Gait parameters	γ	*P*	γ (adjusted for confounders)	*P* (adjusted for confounders)
Gait speed	−0.367	< 0.001	−0.158	0.022
Cadence	−0.237	< 0.001	−0.133	0.055
Stride length	−0.431	< 0.001	−0.243	< 0.001
Stance phase percentage	0.269	< 0.001	0.170	0.014
Foot fall	−0.497	< 0.001	−0.313	< 0.001
pre-swing angle	−0.313	< 0.001	−0.166	0.016
Tinetti gait test	−0.449	< 0.001	−0.177	0.016
Tinetti balance test	−0.489	< 0.001	−0.238	0.001

*The confounders included age, proportion of men, hypertension and stroke, level of blood creatinine, Fazekas score, the number of lacunae, and CMB.*

To identify the effect of BG-EPVS on gait, we took the gait parameters and Tinetti test scores as dependent variables and conducted the multiple linear regression analysis. The results of the multiple linear regression analysis showed that BG-EPVS was an independent risk factor for gait disturbances and balance after adjusting for age, the proportion of male sex, hypertension, and stroke, level of blood creatinine, Fazekas score, the number of lacuna and CMB. The detailed analysis results are presented in Table 4.

**TABLE 4:** Results of multiple linear regression analysis between gait performance and BG-EPVS.

Gait parameters	Model 1		Model 2		Model 3	
			
	β	*P*	β	*P*	β	*P*
Gait speed	−0.294	< 0.001	−0.281	< 0.001	−0.178	0.022
Cadence	−0.183	0.008	−0.180	0.015	−0.167	0.055
Stride length	−0.360	< 0.001	−0.343	< 0.001	−0.247	< 0.001
Stance phase percentage	0.233	0.001	0.241	0.001	0.205	0.014
Foot fall	−0.419	< 0.001	−0.404	< 0.001	−0.346	< 0.001
pre−swing angle	−0.203	0.002	−0.209	0.002	−0.191	0.016
Tinetti gait test	−0.381	< 0.001	−0.352	< 0.001	−0.188	0.016
Tinetti balance test	−0.419	< 0.001	−0.397	< 0.001	−0.255	0.001

*Model 1: adjusted for age and sex. Model 2: model 1 + proportion of hypertension and stroke, level of blood creatinine. Model 3: model 2 + Fazekas score, the number of lacuna, and CMB.*

### Association Between BG-EPVS and Gait Variability

There were no statistical differences in stride length variability [27.6 (13–30.4) vs. 24.3 (11.7–27.7), *P* = 0.08] and stride time variability [11 (8.6–26.4) vs. 12.3 (8.9–30), *P* = 0.374] between the BG-EPVS group and the control group. Point-biserial correlation analysis also showed that BG-EPVS was not related to gait variability (*P* > 0.05).

## Discussion

In the present study, we explored the association between BG-EPVS and gait performance in older individuals with CSVD after adjusting for WMH, CMB, and lacunes. We found that large numbers of BG-EPVS were independently related to gait disturbances, including lower gait speed, shorter stride length, longer stance phase, smaller pre-swing angle, and poorer foot control using a quantitative gait analysis system. In addition, we found that BG-EPVS was also independently related to poor body balance. However, BG-EPVS was not related to gait variability.

The present clinical experience and previous studies indicated that, in subjects with CSVD, symptoms mainly arose in only moderate or severe cases (Baezner et al., 2008; de Laat et al., 2010). For example, gait disturbances were only attributed to moderate or severe WMH and/or the presence of > 3 lacunar infarcts (de Laat et al., 2010). Therefore, in the present study, we classified the subjects with high-grade BG-EPVS into the BG-EPVS group and others were classified into the control group.

Previous studies investigated the relationship between gait performance and other CSVD MRI markers, such as WMH, lacunar infarction, and CMB (Litak et al., 2020). The Radboud University Nijmegen Diffusion tensor and MRI Cohort (RUN DMC) study found that WMH and lacunar infarcts were both independently associated with most gait parameters, and stride length was the most sensitive parameter related to WMH (de Laat et al., 2010). WMH in the basal ganglia and lacunar infarcts in the thalamus were related to a lower velocity. The RUN DMC study also found that a larger number of CMB in the basal ganglia was independently related to a shorter stride length and poorer performance on the Tinetti tests (de Laat et al., 2011b). However, the clinical studies specifically addressing the association between EPVS and gait in patients with CSVD were scarce. Shin et al. (2021) explored the relation between BG-EPVS and EPVS in white matter and motor function in patients with PD. They found that severe BG-EPVS was associated with worse motor symptoms. Chung et al. (2021) investigated the association between BG-EPVS and long-term motor outcomes in patients with PD. They found that the expression of the dopamine transporter was much lower, and the risk of freezing of gait was higher in the PD-EPVS + group. The PD-EPVS + group required higher doses of dopaminergic medications for effective symptom control than the PD-EPVS- group. In the present study, we explored the relationship between BG-EPVS and gait in older patients with CSVD. We found large numbers of BG-EPVS in older patients with CSVD independently related to worse gait performance, similar to the findings in previous studies. Su et al. (2017) investigated the correlation between CSVD burden and motor performance in community-dwelling populations and found that EPVS was not associated with motor performance, a finding that differed from the results of our study. Su et al. (2017) classified patients with EPVS > 5 on the basal ganglia slice containing the maximum number into the EPVS group. The different results might be attributed to the different grouping methods and study populations.

Gait velocity is determined by both the stride length and gait cadence. In the present study, the gait velocity, stride length, and gait cadence were reduced in patients with large numbers of BG-EPVS. However, large numbers of BG-EPVS were only significantly related to gait velocity and stride length and not related to gait cadence after adjusting for WMH, lacunes, and CMB. We propose that stride length may be a more sensitive indicator than gait cadence. Aside from the lower velocity, we found that gait in subjects with severe BG-EPVS was characterized by an increased stance phase percentage, smaller pre-swing angle, reduced footfall, and lower Tinetti balance score, which indicated that severe BG-EPVS is related to poor balance.

The potential pathophysiological mechanisms underlying the association between BG-EPVS and gait are not completely understood. Perivascular spaces are thought to serve as a protolymphatic system and play an important role in maintaining neural homeostasis (Gouveia-Freitas and Bastos-Leite, 2021). Sulcal CSF is cleared through arachnoid granulations or it enters the parenchyma *via* the perivascular spaces, where it combines with interstitial fluid prior to exiting the brain. This perivascular drainage system also allows for the clearance of toxic metabolites within the parenchyma and possibly plays a role in the brain’s immunological response (Iliff et al., 2012). EPVS are thought to be the result of perivascular blockages that disrupt the normal function of perivascular spaces. Although a few EPVS visible on MRI are normal, the presence of many is not normal, and they have been shown to be associated with some age-related disorders, including cognitive dysfunction, Parkinson’s syndrome, WMH, and lacunar infarction (Rudie et al., 2018; Rundek et al., 2019; Gouveia-Freitas and Bastos-Leite, 2021). The basal ganglia–thalamocortical circuit, brain network efficiency, and loss of white matter microstructural integrity are involved in gait impairment in subjects with WMH, lacunes, and CMB (de Laat et al., 2011a; Cai et al., 2021). Recent findings showed that EPVS leads to white matter microstructural damage by causing a glymphatic dysfunction that results in the accumulation of toxic metabolic products. These metabolic products were harmful to the brain microenvironment (Che Mohd Nassir et al., 2021). Therefore, we speculated that large numbers of BG-EPVS may disrupt the functioning of the basal ganglia, brain network, and brain microenvironment and subsequently lead to gait disturbances and poor balance. The hypothesis should be tested and verified with multimodal MRI (e.g., functional MRI and diffusion tensor imaging) in the future.

Some limitations in the present study must be mentioned. First, our study was performed in a single center and the cohort may not represent the general population. Second, this was an observational study, and the causal relationship between BG-EPVS and gait disturbances cannot be established. Third, the mechanisms underlying the association between BG-EPVS and gait disturbances were not explored. A multicenter prospective cohort study using multimodal MRI to explore the mechanisms should be performed in the future. Despite these limitations, our study found that BG-EPVS was associated with gait disturbances in older individuals with CSVD with both quantitative and semiquantitative gait analysis. The novel findings provide some information about the consequences of BG-EPVS and risk factors for gait disturbances in older people.

In summary, we found that large numbers of BG-EPVS were independently related to gait disturbances and poor balance. Patients with severe BG-EPVS had lower gait speed, shorter stride length, longer stance phase, smaller pre-swing angle, and poorer foot control. The causal relationship and mechanisms should be further tested and explored in longitudinal studies in the future.

## Data Availability Statement

The original contributions presented in this study are included in the article/supplementary material, further inquiries can be directed to the corresponding author/s.

## Ethics Statement

The studies involving human participants were reviewed and approved by the Ethics Committee of Beijing Chaoyang Hospital Affiliated to Capital Medical University. The patients/participants provided their written informed consent to participate in this study. Written informed consent was obtained from the individual(s) for the publication of any potentially identifiable images or data included in this article.

## Author Contributions

WH and SY conceived and designed the study. XL and SY participated in the screening participants and data collection. WQ and LY assessed the imagings. SY participated in the data analysis and drafted the manuscript. WH revised the manuscript. All authors read and approved the final manuscript to be published.

## Conflict of Interest

The authors declare that the research was conducted in the absence of any commercial or financial relationships that could be construed as a potential conflict of interest.

## Publisher’s Note

All claims expressed in this article are solely those of the authors and do not necessarily represent those of their affiliated organizations, or those of the publisher, the editors and the reviewers. Any product that may be evaluated in this article, or claim that may be made by its manufacturer, is not guaranteed or endorsed by the publisher.
